# Evaluation of PCR Approaches for Detection of *Bartonella bacilliformis* in Blood Samples

**DOI:** 10.1371/journal.pntd.0004529

**Published:** 2016-03-09

**Authors:** Cláudia Gomes, Sandra Martinez-Puchol, Maria J. Pons, Jorge Bazán, Carmen Tinco, Juana del Valle, Joaquim Ruiz

**Affiliations:** 1 ISGlobal, Barcelona Ctr. Int. Health Res. (CRESIB), Hospital Clínic - Universitat de Barcelona, Barcelona, Spain; 2 Faculty of Health Sciences, School of Medicine, Centro de Investigación de la Universidad Peruana de Ciencias Aplicadas (UPC), Lima, Peru; 3 Dirección Regional de Salud de Cajamarca (DIRESA-Cajamarca), Cajamarca, Peru; 4 Instituto de Investigación Nutricional, Lima, Peru; University of California San Diego School of Medicine, UNITED STATES

## Abstract

**Background:**

The lack of an effective diagnostic tool for Carrion’s disease leads to misdiagnosis, wrong treatments and perpetuation of asymptomatic carriers living in endemic areas. Conventional PCR approaches have been reported as a diagnostic technique. However, the detection limit of these techniques is not clear as well as if its usefulness in low bacteriemia cases. The aim of this study was to evaluate the detection limit of 3 PCR approaches.

**Methodology/Principal Findings:**

We determined the detection limit of 3 different PCR approaches: *Bartonella*-specific *16S rRNA*, *fla* and *its* genes. We also evaluated the viability of dry blood spots to be used as a sample transport system. Our results show that *16S rRNA* PCR is the approach with a lowest detection limit, 5 CFU/μL, and thus, the best diagnostic PCR tool studied. Dry blood spots diminish the sensitivity of the assay.

**Conclusions/Significance:**

From the tested PCRs, the *16S rRNA* PCR-approach is the best to be used in the direct blood detection of acute cases of Carrion’s disease. However its use in samples from dry blood spots results in easier management of transport samples in rural areas, a slight decrease in the sensitivity was observed. The usefulness to detect by PCR the presence of low-bacteriemic or asymptomatic carriers is doubtful, showing the need to search for new more sensible techniques.

## Author Summary

Carrion’s disease is an endemic illness in the Andean valleys of Peru that achieves high mortality rates in the absence of antibiotic treatment. There are three clinical manifestations, febrile acute patients, chronic patients as well as asymptomatic carriers. No effective diagnostic tool exists nowadays leading to misdiagnosis and the perpetuation of the illness. The objective of this study was to determine the detection limit of three PCR approaches both from blood samples as well as from filter papers. Furthermore, the specificity was also accessed. We found that the best PCR approach studied was the amplification of the *16S rRNA* from blood samples with a detection limit of 5 CFU/μL, the same when using dry blood in filter paper, although the obtained bands were not so evident. Present results highlight the need to develop more sensitive techniques able to be used both in rural areas and in the detection of asymptomatic carriers.

## Introduction

*Bartonella bacilliformis* is the etiological agent of Carrion’s disease, an overlooked illness with a lethal febrile stage and a warty phase. Its endemicity is restricted to Peru, Ecuador and Colombia, with some cases having been described in Bolivia and Chile. The transmission is by a sandfly of the genera *Luyzomyia*, mostly *Lutzomyia verrucarum* [1]. The human is the only reservoir known, and in endemic areas about 40% of asymptomatic carriers have been described [2]. In addition, Carrion’s disease-like syndromes have been related to two other *Bartonella* species: *Bartonella rochalimae* and *Bartonella ancashensis* [3–5]. Although its relevance remains uncertain, these species may be an explanation for the Carrion’s disease cases sporadically reported in distant areas such as Guatemala or Thailand [1]. In fact, *B*. *rochalimae* has been isolated worldwide [6,7].

Although the warty phase is easy to diagnose by the clinic manifestations, the initial febrile stage as well as asymptomatic carriers, are often misdiagnosed or non-diagnosed leading to perpetuation of the illness. Correct diagnosis of both acute and asymptomatic carriers is extremely important and adequate treatment is imperative to save lives. In endemic areas the diagnosis is usually made by thin blood smear and/or by clinical data. Despite having a specificity of microscopy of 96%, a low sensitivity of 36% has been described [8]. Moreover, other diseases such as malaria, dengue or tuberculosis that are also present, should be taken into account, since the first symptoms are common and may lead to misdiagnosis and erroneous treatments. All these factors are of enormous relevance since the mortality rates of Carrion’s disease are of 40–85% without treatment [9]. Furthermore, even despite receiving correct treatment the mortality rate is of 10% [10]. A more reliable method is blood culture but this is cumbersome, time-consuming and contaminations have been described in the 7–20% of the cultures [11]. Serologic tests have also been described and show a higher specificity of about 85% for both IgM ELISA and indirect fluorescence antibody test, but are difficult for routine practice [1]. Molecular diagnosis by PCR is probably the easiest way to achieve a more accurate diagnosis in endemic areas, as the equipment required is not as sophisticated or expensive, may be installed in different Health Regional Centers which may provide diagnosis to more peripheral patients, and the personnel may be easily trained in technique management. Several PCR approaches have been described in the literature in the last years [12,13]. However, these studies do not generally involve a large number of samples and additionally, as occurs with the remaining diagnostic tools, they are hampered by the lack of a standard case definition. In any case, PCR approaches have been showed as more effective that optical microscopic [12], being able to diagnostic Carrion's disease patients in acute phase previously classified as negatives by thin blood smear. Nonetheless, a critical issue is the detection limit of these techniques, raising doubts about its usefulness in the detection of low-bacteraemia carriers.

Dried blood spot (DBS) is used for the diagnosis of several infectious diseases [14,15], and has been proposed for use as easy method to transfer blood samples from endemic areas to reference centers in order to carry out molecular techniques for the diagnosis of Carrion’s disease [13]. Therefore, since this illness principally affects children in rural areas, DBS may be an easy solution to both the transportation of samples and for small blood volume collection in the pediatric setting.

The aim of this study was to evaluate the detection limit of three PCR approaches designed to detect *B*. *bacilliformis*, both in blood and filter papers to test their potential use for transferring samples from endemic areas to reference centers.

## Materials and Methods

### Bacterial strain

We used a collection strain of *B*. *bacilliformis* from the Institute Pasteur, CIP 57.20 (NCTC 12136). The strain was grown on blood agar (BD, Germany) at 28°C and 5% CO_2_ until confluent growth.

### Blood samples

To accurately quantify the amount of *B*. *bacilliformis* we used flow cytometry from the Citomics core facility of the Institut d’Investigacions Biomèdiques August Pi i Sunyer (IDIBAPS). For this, one grown agar plate was diluted in appropriate buffer and Perfect-count microspheres were used. Serial dilutions (10^6^ CFU/mL—10 CFU/mL) were made in whole blood provided by the blood bank of the Hospital Clinic.

### Dry blood spots

One-hundred μL of the above mentioned bacterial serial dilutions were transfered to Whatmann 903 filter papers and let dry at least one week at room temperature to mimic the sample transfer conditions in a real scenario.

### DNA extraction

DNA extraction was done from 100 μL blood and from dry blood spots with the Qiamp DNA Mini Kit (Qiagen, Germany), according to the manufacturer’s instructions except that the final elution volume was 100 μL.

### PCR amplification

Fragments of *Bartonella*-specific *16S rRNA*, flagellin (*fla*) genes as well as the variable-intergenic region (*its*) were amplified. The primers used were 5’-CCTTCAGTTMGGCTGGATC-3’ and 5’-GCCYCCTTGCGGTTAGCACA-3’ for *16S rRNA* [16], 5’-ATAGAAAGAGCCTGAATACC-3’ and 5`-TGATGAAGCATGACAGTAACAC-3’ for flagellin and 5’-AGATGATGATCCCAAGCCTTCTGG-3’ [17], and 5’-CTTCTCTTCACAATTTCAAT-3’ [18] for the amplification of variable-intergenic region. The PCRs were performed in a 25-μL total reaction volume with 500 nM forward primer, 500 nM reverse primer, 9,75 μL H_2_O and 5 μL of DNA following the conditions: 30 seconds at 94°C, 30 seconds at 55°C and 2 minutes at 72°C for 30 cycles. A 2% agarose gel stained with Sybr Safe was performed, and the results were visualized with an ImageQuant LAS4000 transiluminator (GE Healthcare Europe GmbH, Barcelona, Spain).

### Detection limit

The detection limit was considered as the lowest dilution at which a positive result was obtained and considering the number of copies of each gene in the *B*. *bacilliformis* genome. All the above mentioned experiments were done in duplicate intra-assay and at two different times.

### Specificity

The specificity was tested by doing the same PCR approaches to other member of the *Bartonella* genus both *in vitro*: *Bartonella elizabethae* (strain 30455), *Bartonella grahamii* (strain 50771), *Bartonella henselae*, *Bartonella koehlerae* (strain 30773), *Bartonella tamiae* (Strain Th307), and *Bartonella vinsonii* subsp. vinsonii (strain 30453), and *in silico* for the remaining 25 recognized species plus *B*. *ancashensis*. In addition other plate-grown bacteremia microorganisms such as *Escherichia coli*, *Pseudomonas* spp., *Shigella* spp., *Klebsiella* spp., *Haemophilus* spp., *Staphylococcus aureus* and *Streptococcus* spp., as well as an intracellular microorganism such as *Ricketsia* spp. and *Brucella melitensis* were also tested.

## Results

When DNA was directly extracted from the blood, the detection limit was 5 CFU/μL for both the *Bartonella*-specific *16S rRNA* and the *fla* genes. Meanwhile, a limit of 500 CFU/μL was obtained on amplification of the *its* region. In the case of DBS, the *Bartonella*-specific *16S rRNA* PCR approach showed the lowest detection limit, which was also of 5 CFU/μL. Concerning dry blood, despite the detection limit being the same for *16S rRNA* and *its*, the sensitivity decreased for *fla* when the detection limit dropped to 500 CFU/μL compared with 5 CFU/μL obtained directly from blood (Table 1). It was of note that fainter bands were always obtained with DBS.

**Table 1:** Detection limit for the 3 PCR approaches studied both for blood samples and dried blood spots.

Blood samples (CFU/μL)			Dried blood spots (CFU/μL)		
*16S rRNA*	*fla*	*its*	*16S rRNA*	*fla*	*its*
5	5	500	5	500	500

Regarding specificity, the *16S rRNA* gene amplifies for all *Bartonella* species (either *in vivo* or *in silico*) but a positive result was also obtained when tested *B*. *melitensis*. The *its* amplification assay was specific for *Bartonella* spp., and no other of the tested microorganisms had a positive PCR. Moreover, the *its* scheme might allow to distinguish between different *Bartonella* spp. by the different amplified size. The *fla* gene amplification was also specific for *Bartonella* species and differentiates between *Bartonella* spp. causing Carrion’s disease (*B*. *bacilliformis*, *B*. *rochalimae* and *B*. *ancashensis)* and the remaining *Bartonella* causing human disease (Table 2) once no amplification was obtained or predicted for the last ones.

**Table 2:** Amplification sizes in different *Bartonella* spp. for each of the three PCR approaches in study.

		PCR approaches (bp)		
Microorganism^1^	Illness^2^	*16S rRNA*	*its*	*fla*
**Carrion’s disease involved *Bartonella* spp.**				
***B*. *bacilliformis***	**Carrion’s disease**	**438**	**545**	**937**
*B*. *rochalimae*	Carrion’s disease ^3^	438	696	974
*B*. *ancashensis*	Carrion’s disease ^4^	438	590	940
**Main *Bartonella* spp. causing human illness**				
*B*. *alsatica*	Endocarditis	438	654	NA
*B*. *clarridgeiae*	Cat scratch disease	438	692	997
***B*. *elizabethae***	**Endocarditis**	**438**	**777**	**NA**
***B*. *henselae***	**Cat scratch disease**	**438**	**719**	**NA**
***B*. *grahamii***	**Retinitis**	**438**	**715**	**NA**
*B*. *quintana*	Trench fever, Cat scratch disease	438	619	NA
*B*. *vinsonii* subsp. berkhoffi	Endocarditis	438	727	NA
*B*. *vinsonii* subsp. arupensis	Bacteremia	438	742	NA
**Other *Bartonella* spp.**^5^				
*B*. *bovis*		438	525	NA
*B*. *capreoli*		438	564^6^	NA
*B*. *coopersplainsensis*		438	701	NA
***B*. *koehlerae***		**438**	**677**	**NA**
*B*. *pachyuromydis*		438	545	NA
*B*. *queenslandensis*		438	715	NA
*B*. *schoenbuchensis*		438	527	1008
*B*. *silvatica*		438	690	NA
*B*. *taylorii*		438	689	NA

^1.^- The 32 currently recognized *Bartonella* species (including the three *B*. *vinsonii* subsp.) plus *B*. *tamiae* were considered.

^2.^- Only the more relevant pathologies have been referenced here.

^3.^- Described as one case of Oroya fever-like in a tourist returning from Peru [4].

^4.^- Described as a cause of Peruvian Wart in children living in an endemic area [3,5].

^5.^- Most of them isolated from animals, and some sporadically reported from human infections. Indicated are only those microorganisms that have the *fla* gene or that the amplified *its* product differing 20 bp or less respecting any of *Bartonella* spp. involved in Carrion’s disease.

^6.^- Uncertain amplification (two gaps close to primer 3’ terminal).

NA: non amplified or non-predicted amplification.

Highlighted in bold when experimental amplification of the three PCR approaches in study were performed.

## Discussion

Carrion’s disease is an overlooked and restricted disease that affects the poorest populations living in remote rural areas, which badly communicated, without equipped laboratories, and with many other illnesses with a common symptomatology [1]. Thus, correct diagnosis of Carrion’s disease is essential, particularly since misdiagnosis is frequent [12, 19]. PCR techniques rank among the most rapid techniques to diagnose *B*. *bacilliformis*. For this reason, the determination of the detection limit of these techniques is extremely important. For this study we have chosen three approaches, the amplification of *16S rRNA*, the hypervariable intergenic transcribed spacer 16S-23S rRNA and the *fla* gene which codes for the flagelin protein of *B*. *bacilliformis*. The amplification of *16S rRNA* has been proposed for Carrion’s disease diagnostic in Peru [12]. All tested *Bartonella* had an amplified product of 438 bp. Moreover, the *in silico* analysis showed that these primers are able to amplify all *Bartonella* spp. Then, this PCR approach may be also useful in other environments to detect and identify other *Bartonella* spp. either combining with sequencing or RFLP.

The *its* amplification permits to differentiate between *B*. *bacilliformis* and *B*. *ancashensis* from the main pathogenic *Bartonella* spp [20]. In fact the *its* region has been used in different studies of *Bartonella* spp [7].

Regarding *fla*, this gene permit to distinguish between the three *Bartonella* causing Carrion’s disease: *B*. *bacilliformis* (940 pb), *B*. *rochalimae* (974 bp) and *B*. *ancashensis* (937 bp) from the remaining *Bartonella* spp. with clinical interest. However, one exception is *B*. *clarridgeiae* (997 bp). Additionally, and *in silico* analysis showed that *B*. *schoenbuchensis* will also results in a positive fragment of 1008 bp.

Our results show that the *Bartonella*-specific *16S rRNA* PCR seems to be the best of the techniques analyzed to detect the presence of *B*. *bacilliformis* in blood samples (5 CFU/μL) since the lowest detection limit was achieved on comparison with *fla* and *its* PCRs. These results are in accordance with Angkasekwinai *et al*. [21], who reported a detection limit of 1 and 10 copies/μL in a loop-mediated isothermal amplification when the detection limit was determined using bacterial genomic DNA alone or in the presence of human plasma respectively. This sensitivity might allow diagnosing the acute cases of Carrion’s disease, in which the mean percentage of infected RBCs is 61% (ranging from 2 to 100%) [22]. Nonetheless, the concomitant use of these PCR approaches will provide information about other *Bartonella* spp. infections.

Filter paper may be an alternative for easy transportation of samples from endemic areas to reference laboratories but the decreasing sensitivity of the results must been taken into account which may lead to the non-detection of cases with a low bacteremia. Although the same detection limit was obtained for *16S rRNA* PCR both directly from blood and filter papers, the bands were fainter in the latter. It is true that 1 week delay in the sample processing could affect the PCR by increasing the detection limit. Nonetheless, in rural settings the transfer of samples to reference centers is associated with bad communications ways, resulting in some days from sample collection to molecular determinations.

None of the non-*Bartonella* microorganisms included in the study were positive when *its* or *fla* PCRs were performed. Nonetheless, when *Brucella* spp. was tested, amplification was obtained to *16S rRNA* PCR. Although this is a limitation, it is need to take into account that a diagnostic should to be performed both in the adequate clinic context and in parallel with other diagnostic tools such as differential PCR for *Brucella* diagnostic when needed [23].

The prevalence of asymptomatic people in endemic areas has already been described by PCR being 0.5% [1]. However, the number of inhabitants previous exposed increases to around 40% when serologic techniques like ELISA or IFA are performed [1]. It is need to take into account that *B*. *bacilliformis* possess tropism for both erythrocytes and endothelial cells, being then present a non-blood circulating bacterial. In the chronic illness stage (verrucuous patients) the sensitivity of the microscopical techniques decreases from the 36% described in the acute phase to less than 10% [24], highlighting the lower blood bacterial carriage and a possible transient bacteremia. Those facts might results in false PCR-negative when the technique is applied in the detection of both verrucous patients and asymptomatic carriers.

It is important to remark that in the last years 2 more sensitive PCR techniques have been described in the literature: qPCR [13] in which 24.6% of DBS samples are positive, as well as a loop-mediated isothermal amplification [21] that achieves good results on analysing *Lutzomyia* samples. However, qPCR requires the expertise of trained personnel and is more expensive and difficult to be implemented. Meanwhile the usefulness of loop-mediated isothermal amplification remains to be validated to detect the presence of *B*. *bacilliformis* in human clinical samples. Enrichment of the sample before conventional PCR has been proposed to increase the positivity by 55% when compared with the original blood samples [25]. However, this enrichment technique results in a 14-days delay in sample processing thereby making it unaffordable for diagnostic purposes.

To conclude, here we show that *16S rRNA* PCR have low cfu detection limit and should be used with special attention to test samples from individuals with clinical suspicion of Carrion’s disease since the applicability to detect healthy carriers is not clear. The use of DBS could facilitate the transfer of samples from rural endemic areas to health facilities, despite the possibility of a small decrease in positivity. It is critical to develop rapid, sensitive and specific techniques which may be applied in endemic rural areas to avoid misdiagnosis and to facilitate the detection of asymptomatic carriers and thereby the decrease the number of *B*. *bacilliformis* cases.
